# Factors influencing metabolic syndrome in adult workers: an analysis of data from the 2022 Korea National Health and Nutrition Examination Survey

**DOI:** 10.7586/jkbns.24.031

**Published:** 2024-11-21

**Authors:** Mi-Kyoung Cho, Bora Kim, Seung-Yeon Kong

**Affiliations:** 1 Department of Nursing Science, Chungbuk National University, Cheongju, Korea; 2 Referral Center, Chungbuk National University Hospital, Cheongju, Korea

**Keywords:** Metabolic syndrome, Occupational groups, Obesity, abdominal

## Abstract

**Purpose:**

This study analyzed the prevalence of metabolic syndrome and influencing factors among adult workers aged 19 to 64.

**Methods:**

Data from the ninth Korea National Health and Nutrition Examination Survey conducted in 2022 were utilized. The sample comprised 685 individuals who had measurements of fasting glucose, triglycerides, high-density lipoprotein cholesterol, blood pressure, and waist circumference, which are necessary to diagnose metabolic syndrome. Data analysis was performed using SPSS 26.0, and complex sample logistic regression was conducted to identify factors influencing metabolic syndrome.

**Results:**

Among the participants, 34.3% were diagnosed with metabolic syndrome. Significant differences were observed in sex, age, region, marital status, regular worker, and physical activity between participants with and without metabolic syndrome (*p* < .05). Factors influencing the prevalence of metabolic syndrome included sex, age (with the 19~29 age group showing a significantly lower risk), physical activity, smoking, and perceived stress levels (*p* < .05).

**Conclusion:**

To prevent metabolic syndrome in adult workers, it is essential to promote physical activity, discourage smoking, and encourage effective stress management.

## INTRODUCTION

Metabolic syndrome (MetS) includes metabolic abnormalities, such as abdominal obesity, hypertension, dyslipidemia, and hyperglycemia, and is a major cause of cardiovascular disease and type 2 diabetes [1]. The prevalence of MetS is rapidly increasing worldwide due to urbanization, aging, and lifestyle changes [1,2]. Approximately one-third of adults in the United States [3] and 24.9% of adults in Korea have MetS [4]. If left unchecked, the risk of severe chronic diseases increases and thus, prevention and management are urgent [5]. In addition to immutable factors such as age and sex [6], MetS is affected by changeable factors such as lifestyle, diet, and physical activity [7].

In particular, adult workers are at a high risk of developing MetS due to job stress, long working hours, irregular lifestyles, and lack of physical activity [8], which negatively affects not only personal health but also professional productivity and economic efficiency [9-11]. Workers aged 30~60 have many risk factors for overweight, obesity, and MetS, mainly long sitting hours and an increased risk due to inappropriate eating habits [10]. In particular, long sitting life, an occupational factor, promotes the occurrence of MetS [12]. Studies have shown that improving eating habits [13] and engaging in regular exercise [14] can reduce the risk of MetS, and recent research has actively analyzed the impact of various risk factors and lifestyle habits on the prevalence of MetS. A comprehensive review of prior research indicates that individuals with MetS-related conditions can effectively manage risk factors through improved eating habits and regular exercise, leading to positive outcomes. While studies related to MetS from the Korea National Health and Nutrition Examination Survey (KNHANES) have examined associations with dietary habits, physical activity, smoking, and drinking, most have focused on the overall population [8], adults aged 19 to 65 [15], or middle-aged individuals [14] with an emphasis on age and sex. Research involving workers has concentrated on specific job-related factors, such as long working hours [8] and shift work [12]. Despite the variety of previous MetS studies, additional research is needed to comprehensively and multi-dimensionally analyze MetS-related factors, incorporating the occupational characteristics and lifestyle factors of adult workers aged 19 to 64. Therefore, analyzing the risk factors for MetS in adult workers and studying the effects of health interventions in the workplace are important for improving public health and workplace productivity.

Therefore, this study aimed to investigate the prevalence of MetS according to the characteristics of adult workers aged 19 to 64, and to examine whether there are differences in the characteristics of the participants based on the presence or absence of MetS. Also, data from the KNHANES was used to explore the relationships between various factors influencing MetS in adult workers. These results can be used as basic data to develop a comprehensive nursing intervention plan to prevent and manage MetS among adult workers.

## METHODS

### 1. Study design

This is a secondary data analysis study that analyzed factors affecting MetS in adult workers using data from the 9th KNHANES in 2022.

### 2. Participants

**Figure 1.**
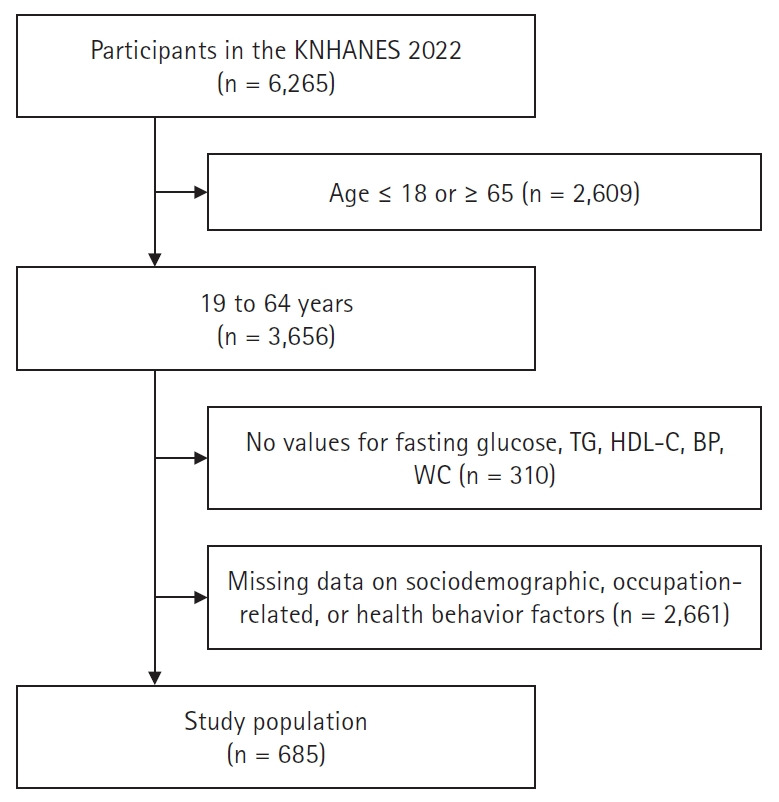
Flow chart of participants, including criteria based on the Korea National Health and Nutrition Examination Survey (KNHANES) in 2022. KNHANES = Korea National Health and Nutrition Examination Survey; TG = Triglycerides; HDL-C = High-density lipoprotein-cholesterol; BP = Blood pressure; WC = Waist circumference.

This study used data from the 9th KNHANES in 2022, provided by the Korea Disease Control and Prevention Agency. The participants of this study were adult workers aged 19~64, and out of the total 6,265 participants, 685 participants were selected as the final analysis participants, who had no missing data in the sociodemographic, occupational, and health behavior characteristics and had fasting glucose, triglycerides, high-density lipoprotein cholesterol, and blood pressure levels and waist circumference measured for MetS (Figure 1).

### 3. Instruments

#### 1) Sociodemographic characteristics

Sociodemographic characteristics of the participants included sex, age, region, education level, marital status, and income level. The participants were aged 19~29, 30~39, 40~49, 50~59, and 60~64 years; education level was categorized as elementary school graduate, middle school graduate, high school graduate, and college graduate or higher; and the region was categorized as dong or eup/myeon. Marital status was categorized as married or single, and income level was categorized as lower, lower-middle, upper-middle, or upper, based on the income quartile standard amount.

#### 2) Occupational characteristics

Occupational characteristics included occupational group, regular workers, weekly working hours, and time spent in a sitting position. The occupational group included managers, experts and office workers, service workers, sales workers, agriculture workers, fishing industry workers, functional workers, mechanical workers, simple labor workers, and military personnel based on the Korean Standard Occupational Classification. However, this study included nine occupations, excluding military personnel who were included under special occupational groups. Workers were divided into regular and non-regular workers. Weekly working hours were divided into three groups as follows: ≤ 40 h, 41~52 h, and > 52 h based on statutory working hours. The time spent sitting was categorized into Q1 (≤ 5 h), Q2 (6~8 h), Q3 (9~11 h), and Q4 (≥ 12 h) by checking the quartiles.

#### 3) Health behavior characteristics

Health behavioral characteristics included diet therapy, frequency of eating out, rate of physical activity, drinking, smoking, health checkups, and perceived stress levels. Diet therapy was categorized as yes or no, and the frequency of eating out was categorized as once a day or more, three times a week or more, 1~2 times a week, 1~3 times a month, or almost never (less than once a month). Physical activity rate was categorized as practicing moderate-intensity physical activity for more than 2 h and 30 min per week, high-intensity physical activity for more than 1 h and 15 min, or a mixture of moderate- and high-intensity physical activity (1 min of high intensity = 2 min of moderate intensity) for the corresponding time of each activity, and not practicing it. Smoking, drinking, and health checkups were categorized as yes or no, and perceived stress level was categorized as feeling it very much, feeling it a lot, feeling it a little, or almost none.

#### 4) Metabolic syndrome

MetS was diagnosed using the diagnostic criteria of the modified National Cholesterol Education Program Adult Treatment Panel III suggested by the American Heart Association/National Heart, Lung and Blood Institute (2005) [15]. If three or more of the five metabolic items (abdominal obesity, high blood pressure, high fasting glucose, high triglycerides, and low high-density lipoprotein cholesterol) were present, it was diagnosed as MetS.

The criteria for abdominal obesity were applied based on the abdominal obesity criteria for Koreans suggested by the Korean Society for the Study of Obesity [16]. Abdominal obesity: waist circumference ≥ 90 cm for men and ≥ 85 cm for women. High blood pressure: systolic blood pressure ≥ 130 mmHg or diastolic blood pressure ≥ 85 mmHg or taking medication. High fasting glucose: Fasting glucose ≥ 100 mg/dL or taking medication. High triglycerides: triglycerides ≥ 150 mg/dL or taking medication. Low high-density lipoprotein (HDL) cholesterol: men < 40 mg/dL, women < 50 mg/dL.

### 4. Data collection

The 9th KNHANES [17] used the 2019 Population and Housing Census to stratify the extraction frame based on city/province, dong/eup/myeon, and housing type (general housing, apartment), and used intrinsic stratification criteria such as residential area ratio, population characteristics of household head age, and single-person household ratio. A total of 6,265 people participated in 192 survey districts from 4,800 households. Health questionnaires, physical examinations, and nutritional surveys were conducted using a mobile medical examination vehicle. A health questionnaire was administered through interviews and self-administered surveys, a nutritional survey through interviews, and a physical examination through direct measurements and specimen analysis. The data for this study were downloaded and used after signing and submitting the “Statistical Data User Compliance” and “Security Pledge” on the KNHANES website (http://knhanes.cdc.go.kr/) [17], followed by the data for the relevant year.

### 5. Statistical analysis

Because the KNHANES data were obtained from multistage stratified cluster sampling, complex sample analysis was performed using strata, clusters, and weights to minimize selection errors. All statistical analyses were performed using IBM SPSS Statistics for Windows (version 26.0; IBM Co., Armonk, NY, USA), and the confidence level was set at 95%, and *p* < .05 was defined as statistically significant. To identify the characteristics and prevalence of MetS in adult workers aged 19~64, and to confirm the relationship between participants' sociodemographic characteristics, occupation-related factors, health behavior factors, and MetS, complex sample cross-analysis and general linear model analysis were performed. Complex sample logistic regression analysis [18] was used to identify the factors affecting MetS in adult workers.

### 6. Ethical considerations

The 9th KNHANES [17] is a statutory survey on health behaviors, prevalence of chronic diseases, and food and nutrition intake, implemented pursuant to Article 16 of the National Health Promotion Act. This survey was conducted with the approval of the Research Ethics Review Committee of the Korea Disease Control and Prevention Agency as a government-designated statistic (approval number 117002), based on Article 17 of the Statistics Act. Since this research was conducted directly by the State for Public Welfare, it was conducted without review by the Research Ethics Review Committee. The data used in this study were raw data from the KNHANES with de-identification measures applied, so that individuals could not be traced. The researcher downloaded the data from the website after receiving approval for its use.

## RESULTS

### 1. Participant characteristics

A total of 685 participants were included in this study, 232 (34.3%) of whom had MetS (Table 1). Of them, 87.2% were men and 12.8% were women, and the average age was 41.85 ± 0.56 years. Of the participants, 85.8% lived in dong, 60.8% had a college degree or higher, 67.8% were married, and 39.0% had a “high” income. Regarding occupational characteristics, 53.4% were managers, experts, and office workers; 63.0% were regular workers; and 37.0% were irregular workers. Approximately 56.2% of the participants worked less than 40 hours per week, and the average working hours were 40.67 ± 0.53. The average sitting time per day was 8.91 ± 0.17 hours. Regarding health behavior characteristics, 74.5% of participants did not follow diet therapy, and 43.7% responded that they ate out more than three times a week. A total of 55.6% of participants practiced physical activity, and drinking and smoking rates were 99.1% and 42.5%, respectively. Of the participants, 78.4% received health checkups and 56.9% felt a little stress (Table 1).

### 2. Prevalence of MetS

The prevalence of the five metabolic items of MetS is shown in Table 2. The prevalence of the five metabolic parameters was as follows: high triglycerides (43.0%), abdominal obesity (42.4%), high fasting glucose (40.2%), hypertension (36.1%), and low HDL cholesterol (16.8%). The number of patients with MetS corresponding to three or more of the five metabolic items was 232 (34.3%). The prevalence of each metabolic item was as follows: low HDL cholesterol (38.8%), high triglycerides (86.2%), abdominal obesity (81.9%), hypertension (71.6%), and high fasting glucose (77.6%). The average waist circumference of the MetS group was 95.74 ± 0.54 cm, which showed more abdominal obesity than the normal group (83.84 ± 0.54 cm). The prevalence of MetS was significantly higher in the group with abdominal obesity (81.9%) than in the normal group (20.1%) (*χ*^2^ = 253.21, *p* <.001). The prevalence rate in the group with hypertension (71.6%) was higher than that in the normal group (19.0%) (*χ*^2^ = 182.14, *p* <.001), and the average fasting glucose in the group with impaired fasting glucose was 114.28 ± 2.14 mg/dL, which was higher than that in the normal group (94.36 ± 0.52 mg/dL). The prevalence rate (77.6%) was also significantly higher than that in the normal group (22.5%) (*χ*^2^ = 189.34, *p* <.001). The prevalence rate of the group with hypertriglyceridemia (86.2%) was higher than that of the normal group (20.5%) (*χ*^2^ = 285.61, *p* <.001), and the prevalence rate of the group with low HDL cholesterol (38.8%) was significantly higher than that of the group without (5.3%) (*χ*^2^ = 131.00, *p* <.001) (Table 2).

### 3. Prevalence of MetS according to participant characteristics

The results comparing the prevalence of MetS according to participants' sociodemographic, occupational, and health behavioral characteristics are shown in Table 1. A total of 232 participants had MetS with a prevalence of 34.3%. Regarding sociodemographic characteristics, the prevalence rate was higher in men (95.3%) than in women (4.7%) (*χ*^2^ = 33.99, *p* <.001), and in terms of age, it was highest in the 50~59 age group (37.5%), and there was a significant difference in the prevalence rate of MetS by age (*χ*^2^ = 49.20, *p* =.001). Additionally, the prevalence of MetS was higher in adults who lived in eups/myeons (20.3%) or were married (83.6%). Regarding occupational characteristics, the prevalence rate of the regular worker group (65.5%) was higher than that of the irregular worker group (34.5%) (*χ*^2^ = 6.55, *p* =.023), but there was no significant difference in other occupational groups, weekly working hours, or time spent in a sitting position. Regarding the health behavior characteristics of participants, the prevalence rate of the group that did not practice physical activity (53.9%) was significantly higher than that of the group that did (*χ*^2^ = 12.03, *p* =.001), but there was no significant difference in other dietary therapies, frequency of eating out, drinking, smoking, health checkups, or perceived stress level.

### 4. Factors affecting MetS in adult workers

The results of the complex sample logistic regression analysis used to identify factors influencing MetS are shown in Table 3. The results of the analysis showed that men were 8.3 times more likely to be exposed to MetS than women (95% confidence interval [CI] = 3.74~18.43, *p* < .001). Compared with the 60~64 age group, the 19~29 age group had a 0.18 times higher risk of MetS (95% CI = 0.06~0.56, *p* = .003), and the group that did not practice physical activity had a 1.74 times higher risk of MetS than the group that did the physical activity (95% CI = 1.15~2.64, *p* = .010). The group that smoked had a significantly higher risk of MetS by 1.49 times (95% CI = 1.03~2.15, *p* = .034) compared with the group that did not smoke. In addition, the group that felt very much stress had a 3.75 times higher risk of MetS than the group that felt a little stress (95% CI = 1.68~8.36, *p* = .001) (Table 3).

## DISCUSSION

This study was conducted to identify the prevalence of MetS in adult workers aged 19~64 years using data from the 9th KNHANES, and to understand the effects of sociodemographic characteristics, occupation-related factors, health behavior factors, and MetS.

In this study, the overall prevalence of MetS was 34.3%, which is slightly higher than the previous findings for South Korea and similar to the prevalence observed in adults in the United States (34.7%) [19]. This suggests that MetS is a widespread issue both in South Korea and globally. The prevalence rate varies from 12.5% to 31.4% across countries [20], which indicates that health policies, socioeconomic factors, lifestyle, and environmental differences between countries play a role in it. In particular, because various factors such as physical activity, smoking, eating habits, and stress have an important influence on the onset of MetS [7,8], prevention and management strategies appropriate to the situation in each country are needed.

MetS generally refers to a condition in which metabolic abnormalities such as obesity, high blood pressure, hyperglycemia, and dyslipidemia are combined, which can increase the risk of cardiovascular disease and diabetes [1]. As a result of this study, the prevalence of MetS in the working group was high at 65.5%, which is likely due to a combination of modern lifestyles, working environments, and lack of awareness of health. It seems that this is because men tend to neglect health care more often than women and engage in risky behaviors such as smoking and drinking, and physical changes that decrease metabolic functions as they get older and increase fat accumulation in the body and insulin resistance increase the risk of developing MetS. It shows that the non-physical group can act as an important factor in increasing the prevalence of MetS, as the full-time working environment is more likely to be exposed to work stress and irregular lifestyles, and the lack of physical activity increases the metabolic function and risk factors such as obesity, high blood pressure, and high blood sugar. Smoking has a negative effect on blood vessels and metabolic health, which can increase the risk of MetS. Stress management is an important factor in preventing MetS because stress has a great impact on metabolic health, such as raising blood pressure, inhibiting blood sugar control, and inducing abdominal fat accumulation. Overall, the causes of increased prevalence of MetS are mainly related to sex differences, age increase, characteristics of regular working environment, lack of physical activity, smoking, and high stress levels. The need for customized health care, physical activity promotion, smoking suppression, and stress management programs according to sex and age is emphasized.

In this study, significant differences between the groups were observed. The prevalence of MetS in men (95.3%) was significantly higher than in women (4.7%), indicating that sex is a critical risk factor for MetS. The prevalence of MetS increased across age groups, reaching its highest level in the 50~59 age group, indicating that MetS is especially common among middle-aged individuals [21,22]. The risk of developing MetS rose with age, consistent with previous findings showing that the older the age, the higher the risk of MetS [21]. In contrast, the prevalence rate was relatively low in younger individuals (19~29 years old). Regarding employment type, the prevalence of MetS was higher among regular workers than among non-regular workers, suggesting that regular workers are more likely to be exposed to factors such as work stress, irregular lifestyles, and long working hours. However, there was no significant difference between occupational factors, such as weekly working hours or time spent sitting, and MetS, which contradicts previous studies showing that long sitting hours promote MetS [12]. This finding suggests that MetS cannot be explained simply by physical inactivity. Even if the sitting time is long, it is possible that intermediate physical activity, dietary habits, and other lifestyle habits, such as stress management, have a greater influence on the occurrence of MetS. In fact, time spent sitting alone may not reflect sufficient physical activity, and previous studies have shown that there is no association between time spent sitting and physical activity levels [23]. This is supported by the fact that the non-physical activity group had a significantly higher risk of developing MetS than the physical activity group. Additionally, individuals who did not engage in physical activity had a significantly higher prevalence of MetS compared to those who engaged in physical activity, highlighting the importance of physical activity in MetS prevention.

In this study, logistic regression analysis confirmed that sex had a significant effect on MetS. Men had an 8.30 times higher risk of MetS than women, supporting that sex differences are important risk factors. Previous studies also confirmed that sex is an important risk factor [24,25], consistent with findings that a significant majority of adult workers are men. This suggests that men, who make up a large portion of the workforce studied, are more susceptible to MetS, which may partly explain the overall higher prevalence observed in this study. Men are generally more likely to neglect health care than women and engage in risky behaviors such as smoking and drinking more frequently, which may increase the risk of MetS [24]. In contrast, the risk of MetS in women is relatively low before menopause due to the protective effect of hormones, especially estrogen [26]. After menopause, however, the risk of MetS tends to increase rapidly as these protective effects diminish [26]. Some studies have shown that the prevalence of MetS in women is higher than that in men [27,28], reflecting the relationship between hormonal changes and MetS according to sex.

Age was also found to significantly affect MetS. In logistic regression analysis, the fact that the risk of MetS in the 19~29 age group was 0.18 times lower than that in the 60~64 age group supported that age is a major risk factor for the development of MetS. These results were attributed to decreased metabolic function and increased insulin resistance, weight gain, and abdominal fat accumulation with age. In addition, as we age, our ability to control blood pressure and blood sugar levels decreases and the likelihood of developing hyperlipidemia increases, and these factors increase the risk of developing MetS. Therefore, it is natural that the risk of MetS increases with age, suggesting that MetS prevention and management programs are needed, especially for adults older than middle age.

In terms of health behavior, a lack of physical activity, smoking, and very much stress levels were found to have significant effects on the prevalence of MetS. Logistic regression analysis revealed that the non-physical activity group had a significantly higher risk of MetS than the physical activity group, emphasizing the importance of physical activity. Physical activity plays an important role in the prevention and management of MetS [14,29,30]. Previous studies have confirmed that not only structured exercise programs but also physical activities through work, place movement, and leisure activities can benefit MetS-related health [31]. Increasing the amount of physical activity in daily life will be effective in improving MetS indicators. Increasing the amount of physical activity in daily life will be effective in improving MetS indicators. Therefore, policies and environmental support are needed to promote physical activity among workers. The smoking group had a significantly higher risk of MetS than the non-smoking group, and smoking was also found to increase the risk of developing MetS. This finding reaffirms the negative effects of smoking on metabolic health [29]. Smoking is the same result of a study that confirmed that it causes hormonal imbalance that increases insulin resistance and affects fat accumulation, which negatively affects MetS indicators as smoking amount rises [30]. Therefore, it is important to reduce smoking or quit smoking to prevent and manage MetS. In particular, it is necessary to promote smoking cessation and educate groups vulnerable to MetS, such as workers and middle-aged people, about the negative effects of smoking on metabolic health. Finally, those who experienced very high levels of stress had a significantly increased risk of MetS compared to those with little stress. The finding that high stress levels increase the risk of MetS suggests that stress has a significant impact on metabolic health. Stress elevates blood pressure, impairs blood sugar regulation, and leads to abdominal fat accumulation through responses of the autonomic and endocrine systems [32], thereby increasing the risk of developing MetS. Chronic stress can trigger risky behaviors such as smoking, drinking, lack of exercise, and irregular eating habits, which may further exacerbate MetS risk. Therefore, workers who sit for long periods, smoke, and experience high levels of stress particularly need workplace environment improvements and tailored management strategies for MetS prevention.

The results of this study emphasize the need for a customized approach that considers factors such as sex, age, physical activity, smoking, and stress to prevent and manage MetS. It is important to develop customized healthcare programs according to sociodemographic and lifestyle characteristics that are expected to reduce the prevalence of MetS and lower the risk of related cardiovascular diseases. This study provides in-depth information on the prevalence of MetS and its related factors, supporting the development of customized health management programs that consider individual characteristics and lifestyles, the establishment of workplace health promotion policies, the use of educational materials, and as foundational data for healthcare professionals and researchers to further investigate specific risk factors or conduct detailed studies. Additionally, the findings of this study can serve as a basis for establishing public health policies at the national level to prevent MetS. Through these applications, this study can contribute to the prevention and management of MetS among adult workers, ultimately enhancing public health and workplace productivity in the long term.

This study had some limitations. First, weekly working or sitting hours did not significantly affect MetS, but this may not reflect the quality and frequency of physical activity. Therefore, an in-depth study is needed to analyze the level of physical activity among professional and job activities, and the effects of policies to encourage physical activity in the workplace. Second, this study analyzed the effects of working hours and occupational characteristics on MetS; however, detailed studies on various occupational groups are needed. Third, the effects of lifestyle habits such as physical activity, smoking, and drinking on MetS are already well known, but studies on how these lifestyle habits interact with job stress or the working environment are insufficient. Therefore, research is needed to analyze the effect of the working environment on lifestyle and to determine how job stress affects the prevalence of MetS. Fourth, the differences in the prevalence of MetS according to sex and age were significant. Through an in-depth analysis of these differences, it is possible to propose a customized management strategy for men and middle-aged people. In particular, research is needed to verify the effectiveness of systematic exercise programs or diets in preventing MetS in middle-aged men. Lastly, only 685 out of 3,656 participants were analyzed after excluding 2,661 participants with missing data on sociodemographic, occupational, and health behavior factors, which may result in biased outcomes due to the large amount of missing data.

## CONCLUSION

The results of this study suggest that the promotion of physical activity, smoking suppression, and effective stress management are essential factors in preventing MetS in adult workers. It is important to reduce physical activity or recommend smoking cessation because smoking is highly associated with MetS. In addition, because stress can worsen MetS, it is necessary to maintain health through effective stress management strategies. This will contribute to improving the health of adult workers and preventing MetS. In future studies, we will study and analyze physical activity, smoking, and stress related to MetS to find a way to understand the occurrence of MetS in depth and effectively prevent and manage MetS.

## Figures and Tables

**Table 1.** Differences in Metabolic Syndrome Prevalence according to Participants' Characteristics (N = 685)

Variables	Categories	Total (n = 685)	Metabolic syndrome		Χ² (*p*)
		n (%)	Yes (n = 232)	No (n = 453)	
Socio-demographic characteristics					
Sex	Men	574 (87.2)	221 (95.3)	353(77.9)	33.99 (< .001)
	Women	111 (12.8)	11 (4.7)	100 (22.1)	
Age (yr)	19~29	115 (16.8)	9 (3.9)	106 (23.4)	49.20 (.001)
	30~39	151 (22.0)	47 (20.3)	104 (23.0)	
	40~49	161 (23.5)	59 (25.4)	102 (22.5)	
	50~59	180 (26.3)	87 (37.5)	93 (20.5)	
	60~64	78 (11.4)	30 (12.9)	48 (10.6)	
		41.85 ± 0.56	46.27 ± 0.75	39.54 ± 0.69	
Region	*Dong*	570 (85.8)	185 (79.7)	385 (85.0)	3.96 (.046)
	*Eup/myeon*	115 (14.2)	47 (20.3)	68 (15.0)	
Education level	Elementary school	18 (1.9)	8 (3.4)	10 (2.2)	1.80 (.928)
	Middle school	26 (3.0)	10 (4.3)	16 (3.5)	
	High school	242 (34.3)	71 (30.6)	171 (37.7)	
	College or above	399 (60.8)	143 (61.7)	256 (56.6)	
Marital status	Married	485 (67.8)	194 (83.6)	291 (64.2)	32.32 (< .001)
	Single	200 (32.2)	38 (16.4)	162 (35.8)	
Income level	Low	36 (5.2)	8 (3.4)	28 (6.2)	0.80 (1.000)
	Middle-low	132 (19.3)	45 (19.4)	87 (19.2)	
	Middle-high	250 (36.5)	86 (37.1)	164 (36.2)	
	High	267 (39.0)	93 (40.1)	174 (38.4)	
Occupational characteristics					
Occupation group	Manager, expert, office workers	350 (53.4)	126 (54.2)	224 (49.5)	18.29 (.240)
	Service, sales workers	115 (16.6)	18 (7.8)	97 (21.4)	
	Agriculture, fishing industry workers	5 (0.5)	2 (0.9)	3 (0.7)	
	Functional, mechanical workers	153 (22.2)	64 (27.6)	89 (19.6)	
	Simple labor workers	62 (7.3)	22 (9.5)	40 (8.8)	
Regular worker	Yes	413 (63.0)	152 (65.5)	261 (57.6)	6.55 (.023)
	No	272 (37.0)	80 (34.5)	192 (42.4)	
Weekly working hours	≤ 40	401 (56.2)	135 (58.2)	266 (58.7)	1.10 (.656)
	41~52	205 (32.1)	67 (28.9)	138 (30.5)	
	> 52	79 (11.7)	30 (12.9)	49 (10.8)	
		40.67 ± 0.53	42.11 ± 0.74	39.92 ± 0.69	
Time spent in sitting position (hr)	≤ 5	148 (19.6)	46 (19.8)	102 (22.5)	3.25 (.344)
	6~8	186 (26.8)	63 (27.2)	123 (27.2)	
	9~11	174 (26.2)	54 (23.3)	120 (26.5)	
	≥ 12	177 (27.4)	69 (29.7)	108 (23.8)	
		8.91 ± 0.17	9.22 ± 0.27	8.75 ± 0.19	
Health behavior characteristics					
Diet therapy	No	501(74.5)	166 (71.6)	335 (74.0)	1.51 (.236)
	Yes	184 (25.5)	66 (28.4)	118 (26.0)	
Frequency of eating out	≥ 1/day	275 (40.7)	95 (40.9)	180 (39.7)	3.83 (.701)
	≥ 3/week	295 (43.7)	102 (44.0)	193 (42.6)	
	1~2/week	83 (12.2)	22 (9.5)	61 (13.5)	
	1~3/month	27 (2.9)	11 (4.7)	16 (3.5)	
	Rarely (< 1/month)	5 (0.5)	2 (0.9)	3 (0.7)	
Physical activity	No	309 (44.4)	125 (53.9)	184 (40.6)	12.03 (.001)
	Yes	376 (55.6)	107 (46.1)	269 (59.4)	
Drinking	No	7 (0.9)	3 (1.3)	4 (0.9)	0.07 (.796)
	Yes	678 (99.1)	229 (98.7)	449 (99.1)	
Smoking	No	393 (57.5)	125 (53.9)	268 (59.2)	1.28 (.314)
	Yes	292 (42.5)	107 (46.1)	185 (40.8)	
Health checkup	No	154 (21.6)	47 (20.3)	107 (23.6)	2.92 (.108)
	Yes	531 (78.4)	185 (79.7)	346 (76.4)	
Perceived stress level	Very much	38 (5.6)	18 (7.8)	20 (4.4)	9.90 (.133)
	A lot	160 (24.1)	56 (24.1)	104 (23.0)	
	A little	394 (56.9)	119 (51.3)	275 (60.7)	
	Almost none	93 (13.4)	39 (16.8)	54 (11.9)	

Values are presented as the mean ± standard error or n (%).

**Table 2.** Prevalence of Each Component of Metabolic Syndrome (N = 685)

Variables	Categories	Total (n = 685)	Metabolic syndrome		Χ² (*p*)
			Yes (n = 232)	No (n = 453)	
Abdominal obesity (WC: men ≥ 90 cm, women ≥ 85 cm)	No	404 (57.6)	42 (18.1)	362 (79.9)	253.21 (< .001)
	Yes	281 (42.4)	190 (81.9)	91 (20.1)	
		87.93 ± 0.47	95.74 ± 0.54	83.84 ± 0.54	
Hypertension (SBP ≥ 130 mmHg, DBP ≥ 85 mmHg)	No	433 (63.9)	66 (28.4)	367 (81.0)	182.14 (< .001)
	Yes	252 (36.1)	166 (71.6)	86 (19.0)	
		119.86 ± 0.61	126.80 ± 1.01	116.23 ± 0.60	
		77.18 ± 0.50	83.26 ± 0.73	74.00 ± 0.51	
High fasting glucose (mg/dL)	< 100	403 (59.8)	52 (22.4)	351 (77.5)	189.34 (< .001)
	≥ 100	282 (40.2)	180 (77.6)	102 (22.5)	
		101.20 ± 0.93	114.28 ± 2.14	94.36 ± 0.52	
High triglycerides (mg/dL)	< 150	392 (57.0)	32 (13.8)	360 (79.5)	285.61 (< .001)
	≥ 150	293 (43.0)	200 (86.2)	93 (20.5)	
		159.90 ± 6.27	245.34 ± 13.09	115.24 ± 4.05	
Low HDL-cholesterol (men < 40 mg/dL, women < 50 mg/dL)	No	571 (83.2)	142 (61.2)	429 (94.7)	131.00 (< .001)
	Yes	114 (16.8)	90 (38.8)	24 (5.3)	
		53.44 ± 0.64	44.94 ± 0.84	57.89 ± 0.76	

Values are presented as the mean ± standard error or n (%). WC = Waist circumference; SBP = Systolic blood pressure; DBP = Diastolic blood pressure; HDL = High-density lipoprotein.

**Table 3.** Factors Affecting Metabolic Syndrome (N = 685)

Variables	Categories	B	SE	OR	95% CI	*p*
Socio-demographic characteristics						
Sex (ref: women)	Men					
Age (yr) (ref: 60~64)	19~29	2.16	0.40	8.30	3.74~18.43	< .001
	30~39	−1.72	0.57	0.18	0.06~0.56	.003
	40~49	−0.34	0.41	0.71	0.32~1.59	.401
	50~59	−0.30	0.36	0.74	0.36~1.51	.406
Region (ref: *eup/myeon*)	*Dong*	0.50	0.39	1.65	0.76~3.54	.201
Education level (ref: college or above)	Elementary school	−0.20	0.23	0.82	0.52~1.29	.391
	Middle school	−0.68	0.70	0.51	0.13~2.02	.333
	High school	−0.45	0.54	0.64	0.22~1.85	.404
Marital status (ref: no)	Yes	−0.32	0.28	0.72	0.42~1.26	.248
Income status (ref: high)	Low	0.11	0.30	1.11	0.61~2.01	.725
	Middle ~low	−0.20	0.64	0.82	0.23~2.92	.756
	Middle ~high	0.29	0.31	1.33	0.73~2.44	.350
Occupational characteristics		0.05	0.22	1.05	0.67~1.64	.822
Occupation group (ref: simple labor workers)	Manager, expert, office workers	−0.04	0.45	0.96	0.40~2.31	.921
	Service, sales workers	−0.78	0.50	0.46	0.17~1.22	.117
	Functional, mechanical workers	0.17	0.42	1.19	0.52~2.74	.681
Regular worker (ref: no)	Yes	0.28	0.25	1.33	0.80~2.20	.270
Weekly working hours (ref: > 52)	≤ 40	0.00	0.30	1.00	0.56~1.79	.997
	41~52	−0.24	0.29	0.79	0.45~1.39	.408
Time spent sitting position (ref: ≥ 12 hours)	≤ 5	−0.30	0.34	0.74	0.38~1.44	.374
	6~8	−0.04	0.28	0.96	0.55~1.66	.876
	9~11	−0.40	0.27	0.67	0.40~1.14	.140
Health behavior characteristics						
Diet therapy (ref: no)	Yes	0.19	0.20	1.21	0.82~1.80	.339
Frequency of eating out (ref: rarely)	≥ 1/day	−1.29	1.09	0.28	0.03~2.39	.240
	≥ 3/week	−1.03	1.10	0.36	0.04~3.12	.349
	1~2/week	−0.91	1.12	0.40	0.04~3.67	.417
	1~3/month	−0.17	1.19	0.84	0.08~8.86	.887
Physical activity (ref: yes)	No	0.55	0.21	1.74	1.15~2.64	.010
Drinking (ref: no)	Yes	0.41	0.81	1.51	0.30~7.50	.611
Smoking (ref: no)	Yes	0.40	0.19	1.49	1.03~2.15	.034
Health checkup (ref: no)	Yes	−0.51	0.28	0.60	0.35~1.04	.070
Perceived stress level (ref: a little)	Very much	1.32	0.41	3.75	1.68~8.36	.001
	A lot	0.48	0.22	1.62	1.06~2.47	.028
	Almost none	0.73	0.25	2.07	1.27~3.38	.004

SE = Standard error; OR = Odds ratio; CI = Confidence interval; ref=reference.

## Data Availability

The data that support the findings of this study are available from the corresponding author upon reasonable request.
